# Supplementary material for: Real-World Experience among Elderly Metastatic Breast Cancer Patients Treated with CDK4/6 Inhibitor-Based Therapy
Source: Cancers (Basel). 2024 Apr 30;16(9):1749. doi: 10.3390/cancers16091749 (PMC11083425; doi:10.3390/cancers16091749)
Supplement: Supplementary file 1 [file cancers-16-01749-s001.zip › cancers-2951827-supplementary.pdf]

Supplemental Figure 1

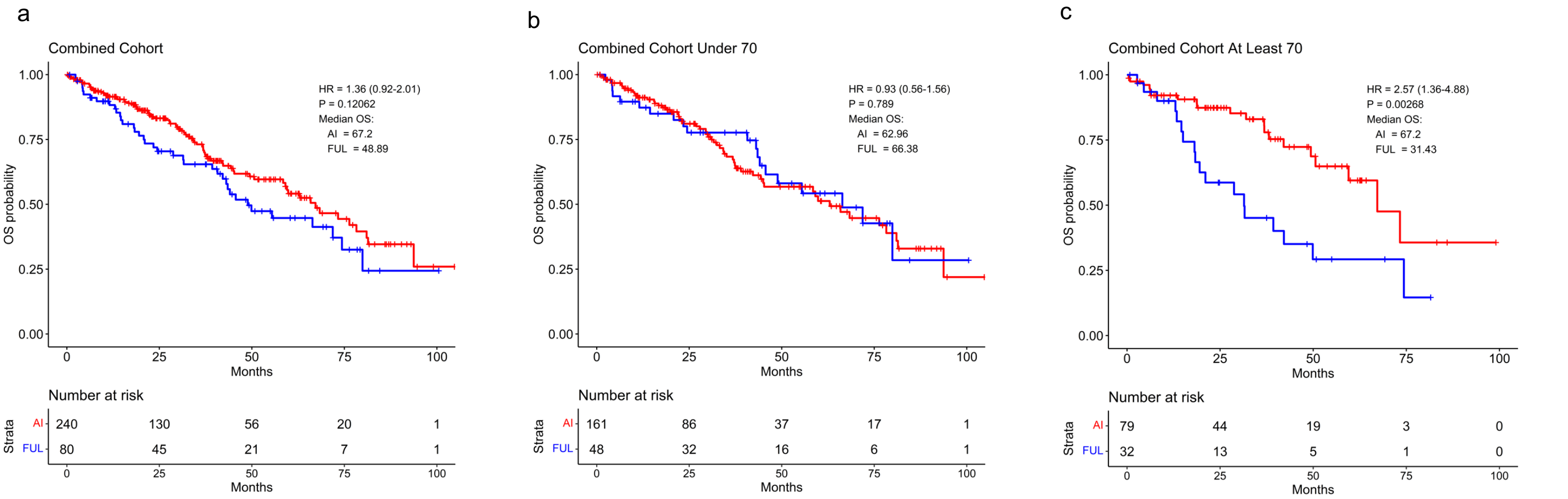

Supplemental Figure 2

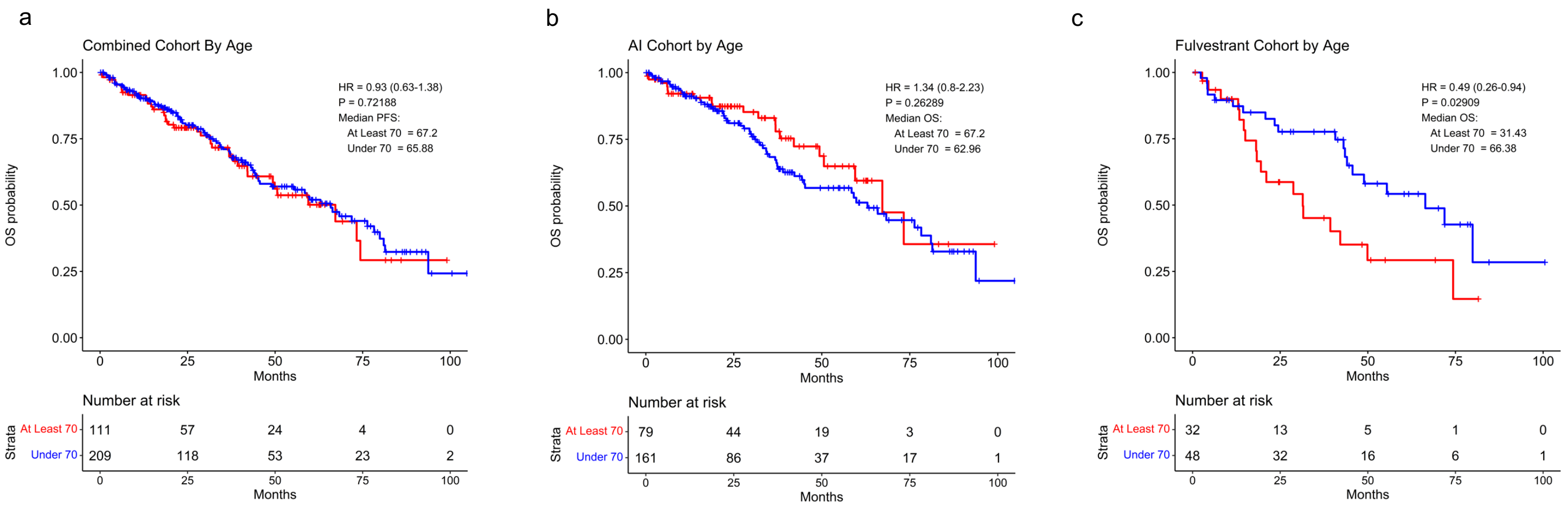

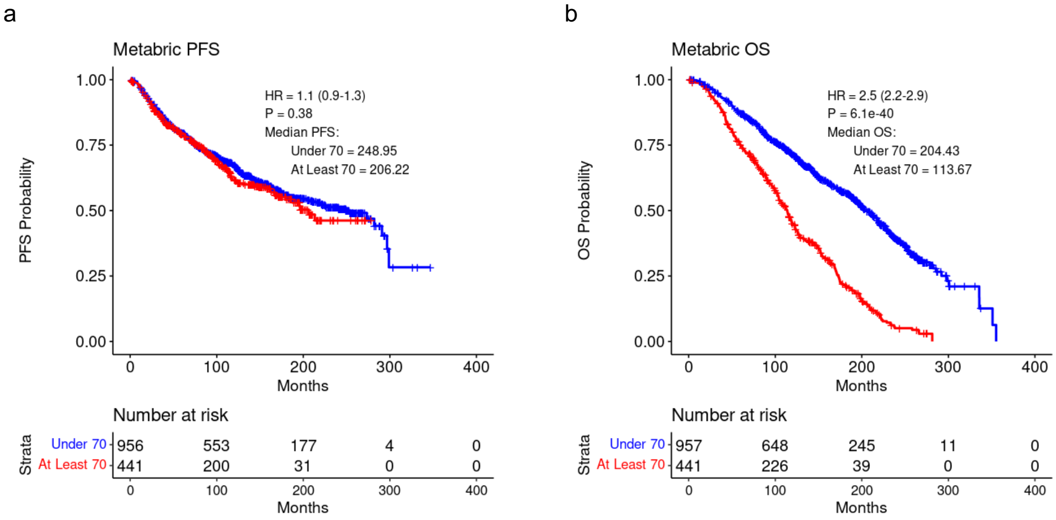
a
Metabric PFS
HR = 1.1 (0.9-1.3)
P = 0.38
Median PFS:
Under 70 = 248.95
At Least 70 = 206.22
PFS Probability
1.00
0.75
0.50
0.25
0.00
0
100
200
300
400
Months
Number at risk
Strata
Under 70
At Least 70
956 553 177 4 0
441 200 31 0 0
Months
b
Metabric OS
HR = 2.5 (2.2-2.9)
P = 6.1e-40
Median OS:
Under 70 = 204.43
At Least 70 = 113.67
OS Probability
Months
Number at risk
Strata
Under 70
At Least 70
957 648 245 11 0
441 226 39 0 0
Months

Supplemental Figure 1. Differences in overall survival of patients taking either aromatase inhibitor or fulvestrant in combination with CDK4/6 inhibitor. (a) Kaplan-Meier plot displaying duration of overall survival (OS) for all patients receiving CDK4/6 inhibitor in combination with either aromatase inhibitor (AI) or fulvestrant (FUL). (b) Kaplan-Meier plot displaying duration of OS for patients under 70 years of age receiving CDK4/6 inhibitor in combination with either AI or FUL. (c) Kaplan-Meier plot displaying duration of OS for patients at least 70 years of age receiving CDK4/6 inhibitor in combination with either AI or FUL. HR: hazard ratio.

Supplemental Figure 2. Differences in overall survival of patients under 70 years of age and at least 70 years of age taking either aromatase inhibitor or fulvestrant in combination with CDK4/6 inhibitor. (a) Kaplan-Meier plot displaying duration of overall survival (OS) for all patients 70 years of age and older or under 70 years of age receiving CDK4/6 inhibitor-based therapy. (b) Kaplan-Meier plot displaying duration of OS for patients 70 years of age and older or under 70 years of age receiving CDK4/6 inhibitor therapy in combination with an aromatase inhibitor. (c) Kaplan-Meier plot displaying duration of OS for patients 70 years of age and older or under 70 years of age receiving CDK4/6 inhibitor therapy in combination with fulvestrant. HR: hazard ratio.

Supplemental Figure 3. Differences in progression-free and overall survival of patients under 70 years of age and at least 70 years of age from the METABRIC database. (a) Kaplan-Meier plot displaying duration of progression-free survival (PFS) for all patients 70 years of age and older or under 70 years of age from the METABRIC database. (b) Kaplan-Meier plot displaying duration of overall survival (OS) for all patients 70 years of age and older or under 70 years of age from the METABRIC database. HR: hazard ratio.
